# Olanzapine Ameliorates Ischemic Stroke-like Pathology in Gerbils and H_2_O_2_-Induced Neurotoxicity in SH-SY5Y Cells via Inhibiting the MAPK Signaling Pathway

**DOI:** 10.3390/antiox11091697

**Published:** 2022-08-29

**Authors:** Md Sadikul Islam, Ha-Young Shin, Yeo-Jin Yoo, Ryunhee Kim, Young-Jin Jang, Md Rashedunnabi Akanda, Hyun-Jin Tae, In-Shik Kim, Dongchoon Ahn, Byung-Yong Park

**Affiliations:** 1 Institute of Animal Transplantation, College of Veterinary Medicine, Jeonbuk National University, Iksan 54596, Korea; 2 Department of Pharmacology and Toxicology, Faculty of Veterinary, Animal and Biomedical Sciences, Sylhet Agricultural University, Sylhet 3100, Bangladesh

**Keywords:** olanzapine, transient ischemia, oxidative stress, neuroprotection, antioxidant, MAPK, differentially expressed genes (DEGs)

## Abstract

Olanzapine (OLNZ) is used to treat psychotic disorders. To look into the neurological basis of this phenomenon, we investigated the neuroprotective effects of OLNZ in gerbils and SH-SY5Y cells. Gerbils were subjected to transient global cerebral ischemia (TGCI) by blocking both common carotid arteries, and OLNZ (10 mg/kg) was injected intraperitoneally. Hydrogen peroxide (H_2_O_2_) was used to induce oxidative-stress-mediated damage in the SH-SY5Y cells. The results indicated that OLNZ administration markedly reduced neuron damage and glial cell triggering within CA1 zone of the hippocampus. We used RNA sequencing to assess the numbers of up-and downregulated genes involved in TGCI. We found that OLNZ treatment downregulated the expression of complement-component-related genes and the expression of mitogen-activated protein kinases (MAPKs) in the hippocampus. In cells, OLNZ co-treatment significantly improved cell viability and reduced lactate dehydrogenase (LDH), and reactive oxygen species (ROS) generation. Expression of antioxidant superoxide dismutase-1,2 enzymes (SOD-1, SOD-2) was also intensely upregulated by OLNZ, while the expression of MAPKs and NF-κB were reduced. Co-incubation with OLNZ also regulated apoptosis-related proteins Bax/Bcl-2 expression. Finally, the results demonstrated that treatment with OLNZ showed neuroprotective effects and that the MAPK pathway could involve in the protective effects.

## 1. Introduction

Cerebral-ischemia-mediated ischemic stroke is a frequently occurring disorder due to sudden obstruction of the blood supply to the cerebrum. Cerebral ischemic injury leads to several cellular alterations that are influenced by free radical generation, excitotoxicity, and metabolic disorder [1,2,3]. Neuronal cell death and glial cell activation in the ischemic penumbra are considered the main cellular features of cerebral ischemic insult that could be targeted by administration of neuroprotectants within 6 h of induction of stroke [4].

The balance between intracellular oxidation and reduction (i.e., redox status) is an important physiological parameter of cells [5]. When this balance is altered by abnormal stimuli, cells may undergo oxidative stress, leading to the production of a huge amount of reactive oxygen species (ROS) by mitochondria [6]. Excessive production of ROS (including the superoxide radical, O_2_·^−^) contributes to neuronal cell damage by disrupting cell membranes, damaging DNA, and triggering cellular apoptosis [7,8,9]. Antioxidant superoxide dismutase enzymes (SODs) are able to convert O_2_·^−^ into hydrogen peroxide (H_2_O_2_) and oxygen (O_2_). Hence, SODs are considered important therapeutic targets for the management of oxidative stress by preventing ROS generation [10].

Mitogen-activated protein kinases (MAPKs) play an essential part in transforming extracellular signals towards a variety of cellular actions, together with cellular progress, migration, propagation, diversity, and cell death. In neuroblastoma cells, several MAPKs (ERK1/2, JNK, P38) can be activated by H_2_O_2_-induced oxidative stress [11,12]. JNK and p38 are two of them that work in a cellular framework and in specific ways to integrate responses at multiple programs, whether transcription-based or not, and finally activate caspase-dependent death [13]. Similarly, ERK activation modulates apoptosis through stimulation of mitochondrial cytochrome *c* release [14]. Cytochrome *c* release can also be stimulated by pro-apoptotic Bax proteins that mediate mitochondrial permeabilization and induce apoptosis [15]. In addition, the transcription factor nuclear factor kappa B (NF-κB) promotes the production of pro-inflammatory cytokines and chemokines. [16]. Under stressful conditions, the NF-κB pathway is stimulated, which encourages apoptosis [17,18].

Most complement components are produced in the liver, excess deposition of complement components is found in apoptotic neurons in the brain following ischemic stroke [19,20,21]. The complement system increases neuronal damage by activating brain microglia or the impulsive formation of membrane assault complexes with concurrent neuronal lysis [20,22,23]. Therefore, blocking complement cascade activation could be a promising clinical therapeutic target for stroke [24].

A second-generation atypical antipsychotic drug called olanzapine (OLNZ) works by binding to the receptors for dopamine (D1 to D5), serotonin (5-HT_2A_ to 5-HT_2C_), muscarinic choline (M_1_ to M_5_), 1-adrenergic, and also histamine (H1) [25]. It is effective in schizophrenia and bipolar disorder, including mixed or manic episodes [26]. OLNZ has been discovered to have a strong antioxidant effect [27,28]. OLNZ has a controversial effect on the upregulation and downregulation of ERK1/2, JNK, and P38 proteins [29,30]. Moreover, OLNZ has shown neuroprotective effects in PC12 cells [31] and rat hippocampal neurons [32], stimulates neurogenesis in the rat brain [33] and reduces damage in the mouse brain after focal cerebral ischemia [34]. But the mechanism of neuroprotection by OLNZ is not fully understood. The role of OLNZ by inhibiting the activation of MAPK family proteins in ischemic stroke has not been studied yet. Therefore, the objective of this study was to investigate the neuroprotective effects and related mechanisms of OLNZ in transient global ischemia in gerbils and in the neurotoxicity of SH-SY5Y cells.

## 2. Materials and Methods

### 2.1. Animals

Thirty male Mongolian gerbils (24–25 weeks old) was used for this experiment. Animal welfare legislation passed by the Institutional Animal Care and Use Committee (approval no. CBNU-2020-003) of the Jeonbuk National University Laboratory Animal Center in South Korea was followed. All animals were provided with all required facilities including an adequate number of cages, sufficient food and water, suitable temperature (23 ± 2 °C) and humidity (35–60%), and a 12 h light cycle. One week before the experiments, the experimental animals were moved into the laboratory environment.

### 2.2. Experimental Animal Groups and OLNZ Treatment

**Figure 1:**
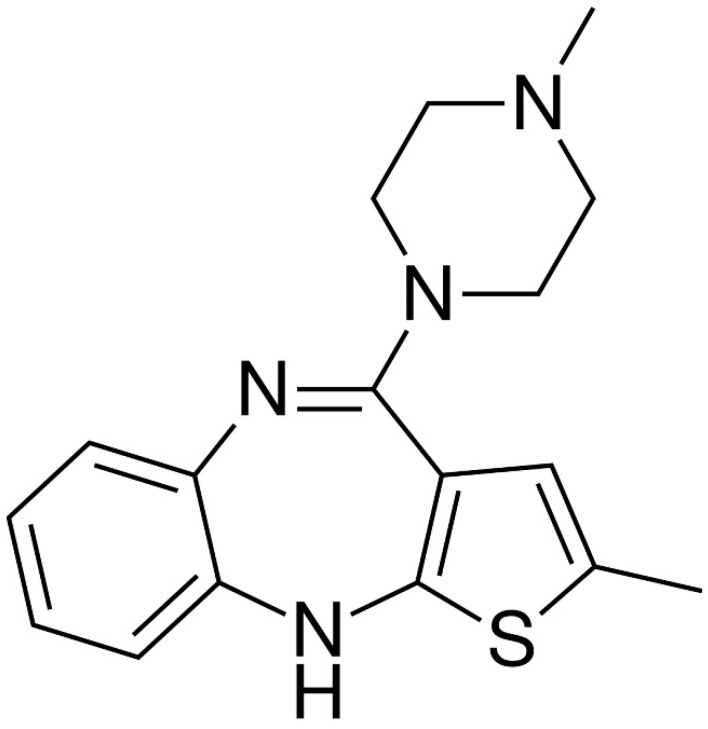
Chemical structure of olanzapine.

Experimental gerbils (*n* = 30) were sorted into three groups: (1) Sham group, which underwent a sham operation; (2) Transient ischemia (TI) + saline group (Figure 1), which was subjected to TI for 5 min and orally administered saline, and (3) TI + OLNZ group, which was treated to TI for 5 min and got a single intraperitoneal injection of 10 mg/kg body weight (Cayman Chemical Company, Ann Arbor, MI, USA).

### 2.3. Method of TI Induction in Gerbils

We implemented a technique to induce TI in gerbils that was described previously [35,36]. In brief, inhalation anesthesia was implemented with a combination of nitrous oxide, oxygen, and isoflurane (68%, 32%, and 2.5%, respectively). Then, the surgical site (midline of the neck) was cleaned, and an incision was made to expose the bilateral common carotid arteries (BCCA). The BCCA were isolated from surrounding muscle and nerve fibers and blocked for 5 min using aneurysm clips (Yasargil FE 723K, Aesculap, Tuttlingen, Germany). Immediately after the obstruction period, OLNZ was injected intraperitoneally (10 mg/kg body) in the TI + OLNZ treatment group.

### 2.4. Tissue Processing for Histological Study

As described in a previously established investigational procedure [35], 5 days after induction of TI, all gerbils in the experiment were sacrificed using 30% urethane. Immediately following euthanasia, intracardial perfusion by 0.1 M PBS, pH 7.4 followed, using 4% paraformaldehyde (PFA) for tissue fixation. All brains were carefully removed from the brain cavity in an intact state and transferred to a conical tube containing 4% PFA for 12 h. After the fixation period, the 4% PFA was changed, and 30% sucrose solution was added overnight. A cryostat (CM1900 UV, Leica, Wetzlar, Germany) device was used to section the brain into 30-µm-thick sections.

### 2.5. Cresyl Violet Staining

To assess neuronal cell destruction induced by TI in the brain, cresyl violet (CV) staining was implemented. Brain sections were placed on pre-coated gelatin slides. Cresyl violet acetate (Sigma, St. Louis, MO, USA) subjected to solved in distilled water (DW) and glacial acetic acid. Slides were dipped into the CV solution for staining and successively submerged into increasing concentrations of ethanol for dehydration. The stained slides were observed under a microscope.

### 2.6. Fluoro-Jade B Histofluorescent Stain

Degenerating neurons in the brain sections was assessed using Fluoro-Jade B (F-J B) staining, as described previously [37]. Briefly, slides of hippocampal sections were immersed in a 0.06% potassium permanganate solution (dissolved in DW) for 20 min. Subsequently, the slides were washed with DW and then dipped into a 0.0004% solution of Fluro-Jade B (Histochem, Jefferson, AR, USA) (dissolved in DW and acetic acid) for 45 min. The tissue sections were dehydrated in an oven (60 °C) and mounted by dibutyl phthalate polystyrene xylene (DPX; Sigma, St. Louis, MO, USA).

### 2.7. Immunohistochemistry

As reported earlier [38], TI-induced immunoreactive pyramidal neurons and activated glial cells in the CA1 domain of the hippocampus were evaluated using immunohistochemistry (IHC). Briefly, to block endogenous peroxidase, cryo-sections of the brain were dipped into a 0.3% hydrogen peroxide (H_2_O_2_) solution. The brain sections were then blocked using 5% normal goat serum for GFAP, 4HNE and Iba-1 or horse serum for NeuN. Blocks of brain tissue were incubated with a variety of antibodies overnight at 4 °C, together with rabbit anti-GFAP (1:1000, Gene tex, Irvine, CA, USA), mouse anti-NeuN (1:800, Chemicon International, Temecula, CA, USA), rabbit anti-Iba-1 (1:1000, Gene tex, CA, USA), and rabbit anti-4HNE (1:800, Abcam, Cambridge, UK), to identify activated astrocytes, mature neurons, and microglia, respectively. The brain sections were then treated with secondary antibodies (Vector Laboratories Inc., Newark, CA, USA), Vectastain ABC (Vector Laboratories Inc.), and diaminobenzidine chromogen, and coverslips were glued with Canada balsam (Kanto Chemical, Tokyo, Japan).

### 2.8. RNA Isolation and Whole Transcriptome Sequencing (RNAseq)

Total RNA isolation from the hippocampal area of the brain (*n* = 3/group) and cells was executed using RiboEx™ total RNA isolation kits (GeneAll, Seoul, Korea), respectively, followed by manufacturer’s indications. A Biospec-nano spectrophotometer was used to determine the quantity and quality of retrieved RNA (Shimadzu Biotech, Tokyo, Japan). Oligo (dT) magnetic beads were used to purify and make a fragment of mRNA molecules from total RNA. The fragmented mRNAs were used to synthesize cDNAs, and cDNA libraries were amplified using a polymerase chain reaction (PCR). The cDNA libraries were measured by an Agilent 2100 BioAnalyzer (Agilent, Santa Clara, CA, USA) and KAPA library quantification kit (Kapa Biosystems, Wilmington, MA, USA), respectively. Finally, RNA sequencing (*n* = 1/group) was completed in a paired-end configuration (2 × 150 bp) using an Illumina Novaseq 6000 (Illumina, San Diego, CA, USA).

### 2.9. Quantitative Real-Time PCR (qPCR) Analyses

To create cDNA (*n* = 3/group), total RNA was extracted and measured. Then, real-time PCR was performed using a Thermal Cycler Dice^®^ Real-Time System III (Takara, Shiga Japan) and TB Green Premix Ex Taq as the real-time PCR master mix. Subsequently, primers were used to perform RT-PCR (Table 1).

### 2.10. Cell Culture and Cell Viability Assay

The Korean Cell Line Bank provided the human neuroblastoma cell line SH-SY5Y. The cells were cultured in equal parts of EMEM (ATCC 30-2003, Manassas, VA, USA) and F12 medium (Gibco, Waltham, CA, USA). To measure the cell viability, the 3-(4,5-dimethylthiazol-2-yl)-2,5-diphenyltetrazolium bromide (MTT) test (Sigma-Aldrich, St. Louis, MO, USA) was utilized [39]. The SH-SY5Y cells were incubated with OLNZ (1, 5, 25, or 100 µg/mL) for 2 h before induction of cytotoxicity by adding 300 µM H_2_O_2_ and co-culturing for another 24 h. After adding the MTT solution, the formation of blue color formazone crystals was detected in each well. A VersaMax™ microplate reader (Molecular Devices, San Jose, CA, USA) was employed to measurement the absorbance at 570 nm.

### 2.11. LDH and ROS Determination Test

Generation of LDH was estimated via an LDH cytotoxicity measurement kit, as directed by the manufacturer (Takara, Shiga, Japan). SH-SY5Y cells subjected to cultured overnight in 96-well plates, pretreated with OLNZ for 2 h with various doses, and then co-cultured with H_2_O_2_ for another 24 h. The relative concentration of LDH from the supernatant of the cells was evaluated by determining the absorbance at 490 nm with an automated microplate reader.

Intracellular ROS generation was distinguished using a fluorometric intracellular ROS detection Kit (Sigma-aldrich, St. Louis, MO, USA). SH-SY5Y cells were incubated overnight in 96-well plates, pretreated with OLNZ for 2 h with various doses, and then co-cultured with H_2_O_2_ for another 12 h. Hanks’ Balanced Salt Solution (HBSS) was used for washing cells. After adding the master reaction mix (100 µL/well), the cells were incubated in the incubator (5% CO_2_, 37 °C) for 30 min. Then, we measured the fluorescence intensity (λ_ex_ = 640/λ_em_ = 675 nm).

### 2.12. Western Blot Analyses

RIPA buffer (Biosesang, Gyeonggi-do, South Korea), and T-Per tissue protein extraction reagent (Thermo Scientific, Rockford, LA, USA) was applied to extract proteins from SH-SY5Y cells and the hippocampal area of brain tissue, correspondingly. Lysed cells were centrifuged at 13,000× *g* for 15 min after being sonicated, and the supernatant was collected. Using a PierceTM BCA protein assay reagent (Thermo Scientific, Rockford, IL, USA), the total protein content of lysed cells was calculated. Then, protein samples were separated using 10–12% sodium dodecyl sulfate-polyacrylamide gel electrophoresis (SDS-PAGE) and added to transfer in a nitrocellulose membrane. To prevent nonspecific binding of primary and secondary antibodies, the membranes were blocked with 5% bovine serum albumin (BSA; Sigma, St. Louis, MO, USA) for 2 h at room temperature. Primary antibodies were mixed with 5% BSA and Tris-buffered saline with Tween^®^-20 (TBST). Membranes were incubated with primary antibodies overnight, and then secondary antibodies were added for 2 h (goat anti-rabbit IgG-HRP; Santa Cruz Biotechnology, Inc. Dallas, TX, USA). Finally, membranes were placed in an ImageQuant™ LAS-500 image system (GE Healthcare, Little Chalfont, UK) to capture band images.

### 2.13. Statistical Analyses

In this experiment, all the measurements and comparative analyses among the groups were analyzed using Graph Pad Prism version 5.0 (Graph Pad Software, Inc., San Diego, CA, USA) and are presented as mean ± standard error (SEM). Analysis of variance (ANOVA) and subsequent Tukey’s post hoc tests were used for the statistical analyses. A minimum *p*-value of 0.05 was deemed significant.

## 3. Results

### 3.1. Neuroprotective Effects of OLNZ against TI-Mediated Neuronal Cell Death

**Figure 2:**
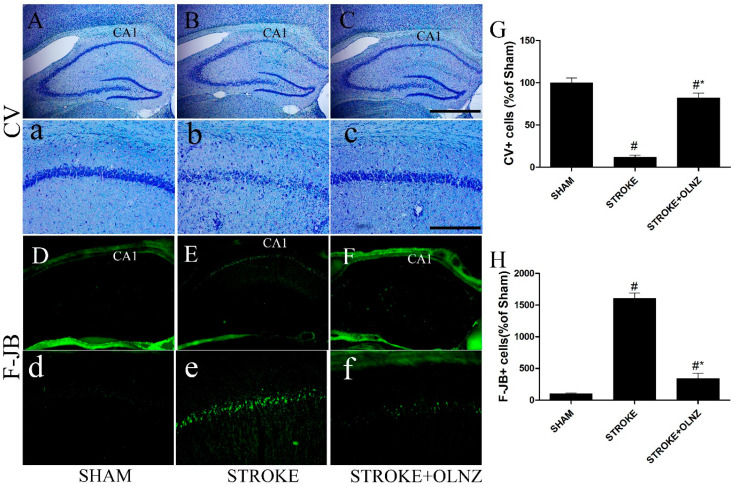
Role of OLNZ in CV positive neuronal cells and F-J B histofluorescence neuronal cells in the hippocampus of CA1 domain. Identification of CV positive cells in the sham group (**A**) 10× and (**a**) 200×, TI-mediated ischemic stroke group (**B**) 10× and (**b**) 200×, and TI-mediated ischemic stroke + OLNZ (10 mg/kg) group (**C**) 10× and (**c**) 200×. F-J B histofluorescent of the CA1 domain in the sham group (**D**) 10× and (**d**) 200×, TI-mediated ischemic stroke group (**E**) 10× and (**e**) 200×, and TI-mediated ischemic stroke + OLNZ (10 mg/kg) group (**F**) 10× and (**f**) 200×. In the TI-mediated stroke gerbils, the number of CV positive cells in the CA1 domain was reduced compared with the sham group, although a greater number of F-J B+ degenerative cells were distinguished in the hippocampus of the ischemic group compared to both the sham and TI-induced stroke + OLNZ treatment groups. (**G**) Graphs represent the relative numbers of CV+ and (**H**) F-J B+ cells. Data were expressed as the mean ± SEM, *n* = 5 in each group. After performing one-way ANOVA with Tukey’s post hoc tests, *p* < 0.05 for comparisons of the treatment groups with the sham group (#) and *p* < 0.05 for comparisons with the TI-induced stroke group (*) were considered significant.

Following obstruction of the BCCA, neuronal damage in the CA1 domain of the hippocampus was observed by histological analysis of selected CV-stained sections in all three groups. Examination of the CV-stained cells revealed that induction of TI significantly reduced the number of live neuronal cells in the pyramidal layer in the CA1 domain (Figure 2B,b), compared with the sham group. Intraperitoneal (I/P) injection of OLNZ (*n* = 5) immediately after induction of TI resulted in a greater number of large pyramidal-shaped CV-positive cells in the CA1 domain, compared to the TI-induced group (Figure 2C,c). Cells that were positive for the neuronal degenerative marker F-J B+ could not be distinguished in the group’s hippocampus either low or high magnification (Figure 2); however, in the TI-induced stroke group, the CA1 area contained large numbers of F-J B+ degenerative cells (Figure 2E,e). In contrast, the expression pattern and the number of F-J B+ cells were notably diminished in the TI-induced stroke + OLNZ treatment group (Figure 2F,f) 6 days after induction of TI.

**Figure 3:**
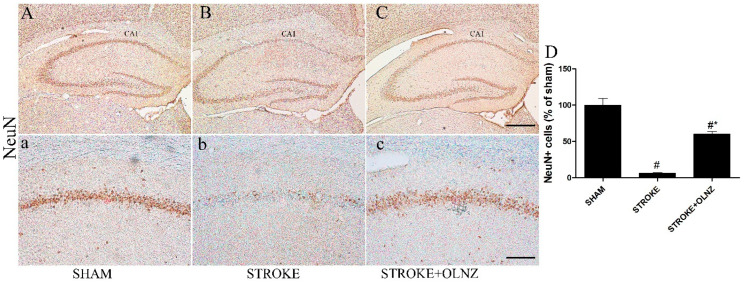
Role of OLNZ in NeuN immunoreactive cells in the CA1 area of the hippocampus. Identification of NeuN+ cells in the sham group (**A**) 10× and (**a**) 200x, TI-mediated ischemic stroke group (**B**) 10× and (**b**) 200x, and TI-mediated ischemic stroke + OLNZ (10mg/kg) group (**C**) 10× and (**c**) 200x. A small quantity of NeuN immunoreactive cells was superficially noticeable in the TI-mediated stroke animals in comparison with the OLNZ-treated animals. (**D**) The graph signifies the relative numbers of immunoreactive NeuN+ cells. Data were expressed as the mean ± SEM, *n* = 5 in each group. After performing one-way ANOVA with Tukey’s post hoc tests, *p* < 0.05 for comparisons of the treatment groups with the sham group (#) and *p* < 0.05 for comparisons with the TI-induced stroke group (*) were considered significant.

Immunohistochemistry (IHC) using NeuN immunoreactivity was performed to identify the nuclear protein expressed by neuronal cells in the CA1 area of the hippocampus (Figure 3). Few NeuN+ neuronal cells were found under either low or high magnification in the TI-induced stroke group (Figure 3B,b), in contrast to the sham group. A single dose of OLNZ (10 mg/kg) significantly increased the NeuN immunoreactive cells in the hippocampus, as confirmed by the well-stained pyramidal-shaped nucleus of the neurons with lighter staining of the cytoplasm (Figure 3C,c).

### 3.2. Neuroprotective Effects of OLNZ by Inactivation of TI-Induced Neuroglia Cells

**Figure 4:**
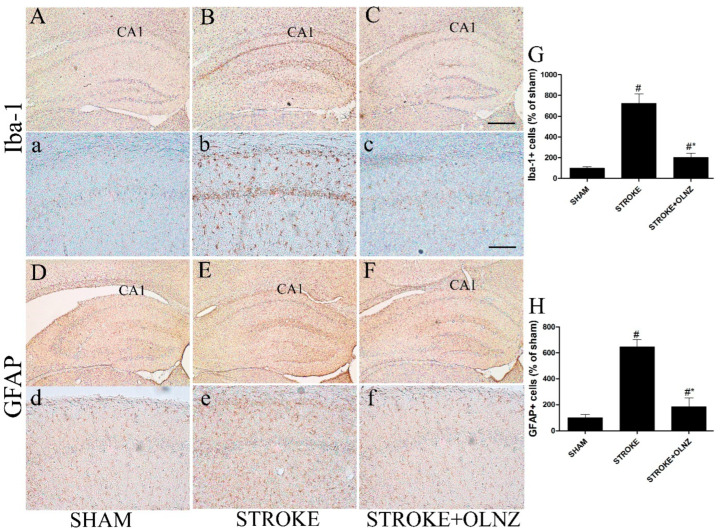
Role of OLNZ in Iba-1-immunoreactive microglia and GFAP-immunoreactive astrocyte cells in the CA1 area of the hippocampus. Identification of Iba-1+ neuronal cells in the sham (**A**) 10× and (**a**) 200×, TI-mediated ischemic stroke (**B**) 10× and (**b**) 200×, and TI- mediated ischemic stroke +OLNZ (10 mg/kg) (**C**) 10× and (**c**) 200× groups and of GFAP immunoreactive astrocytes in the CA1 region in the sham (**D**) 10× and (**d**) 200×, TI- mediated ischemic stroke (**E**) 10× and (**e**) 200×, and TI- mediated ischemic stroke +OLNZ (10 mg/kg) (**F**) 10× and (**f**) 200× groups. In the TI- mediated stroke group, Iba-1+ cells were notably augmented and GFAP+ cells exhibited bigger and denser processes in the CA1 area compared with OLNZ treatment groups. (**G**) Graphs signify the relative numbers of Iba1+ and (**H**) GFAP immunoreactive cells. Data were expressed as the mean ± SEM, *n* = 5 in each group. After performing a one-way ANOVA with Tukey’s post hoc test, *p* < 0.05 for comparisons of treatment groups with the sham group (#) and *p* < 0.05 for comparisons with the TI-induced stroke group (*) were considered significant.

The activation status of both astrocytes and microglial cells was assessed either by immunoreactivity of GFAP+ or Iba-1+ cells in the hippocampus of all three groups after 5 days of TI induction (Figure 4). All TI-induced animals exhibited GFAP+ and Iba-1+ cells in the entire focus of the hippocampus. Neither the sham group nor the OLNZ treatment group showed expression of GFAP+ or Iba-1+ cells, even in the most vulnerable area of the hippocampus (Figure 4).

### 3.3. Neuroprotective Effects of OLNZ by Inhibiting Oxidative Stress

**Figure 5:**
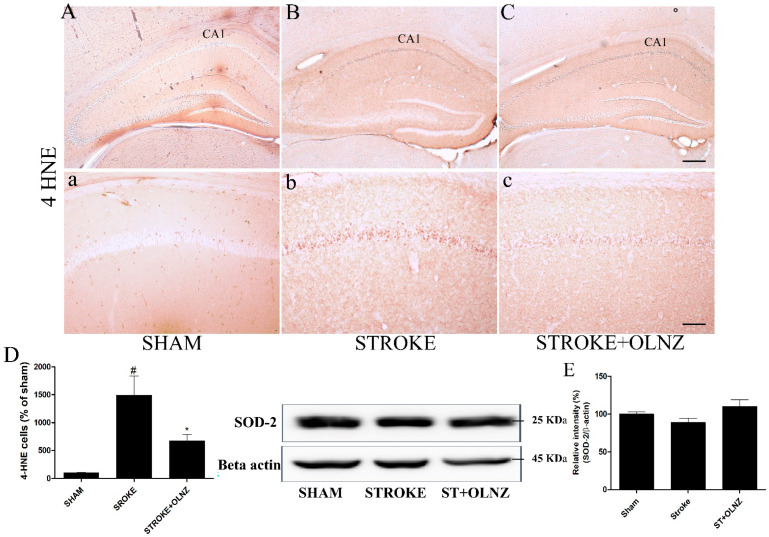
Role of OLNZ in 4-HNE immunoreactive cells in the CA1 area of the hippocampus. Identification of 4-HNE cells in the sham group (**A**) 10× and (**a**) 200×, TI-mediated ischemic stroke group (**B**) 10× and (**b**) 200×, and TI-mediated ischemic stroke + OLNZ (10 mg/kg) group (**C**) 10× and (**c**) 200×. (**D**) The graph signifies the relative numbers of immunoreactive 4-HNE cells. Effects of OLNZ on antioxidant enzyme (SOD-2) protein expression in hippocampal tissue. The expression of (%) (**E**) SOD-2 proteins was increased after treatment with OLNZ. Data were expressed as the mean ± SEM, *n* = 3 in each group. After performing one-way ANOVA with Tukey’s post hoc tests, *p* < 0.05 for comparisons of the treatment groups with the sham group (#) and *p* < 0.05 for comparisons with the TI-induced stroke group (*) were considered significant.

Oxidative stress-induced free radicles attack lipids that provoked lipid peroxidation [40]. Lipid peroxidation marker 4-HNE immunoreactivity was significantly increased in CA1 pyramidal neurons of the TI-induced group (Figure 5B,b) and decreased by OLNZ treatment (Figure 5C,c) after TI. We measured the expression of free radical scavenging enzyme (SOD-2) proteins in the gerbil’s brain by immunoblotting analysis (Figure 5E). SOD-2 expression was decreased in hippocampal neurons of the TI-induced group and increased by OLNZ treatment after TI (not significantly).

### 3.4. Neuroprotective Effects of OLNZ by Blocking MAPK Pathway in the Hippocampus

**Figure 6:**
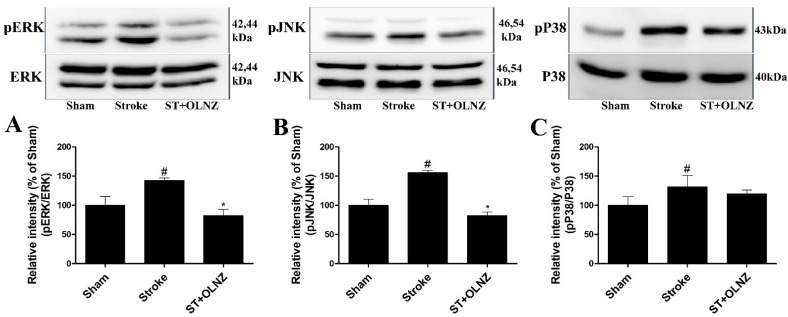
Protective role of OLNZ against the TI-mediated phosphorylation of ERK, JNK, p38 protein in the brain. The expression (% of sham) of (**A**) pERK, (**B**) pJNK proteins decreased significantly, and (**C**) pP38 proteins also decreased but not significantly after treatment with OLNZ in TI-induced gerbil hippocampus. Data were expressed as the mean ± SEM, *n* = 3/group. After performing a one-way ANOVA with Tukey’s post hoc test, *p* < 0.05 for comparisons of treatment groups with the sham group (#) and *p* < 0.05 for comparisons with the stroke group (*) were considered significant.

To investigate the possible pathways involved in OLNZ-mediated neuroprotection, we measured the activation of proteins associated with neuronal cell death, specifically the regulation of MAPK cascades. Experimental results revealed that activation of MAPK (ERK1/2, JNK, and p38) family proteins were notably augmented in the hippocampus after induction of TI, but ERK1/2 and JNK were significantly reversed by treatment with OLNZ (Figure 6).

### 3.5. Effects of OLNZ on DEGs Involved in the Ischemic Stroke Response in the Hippocampus

**Figure 7:**
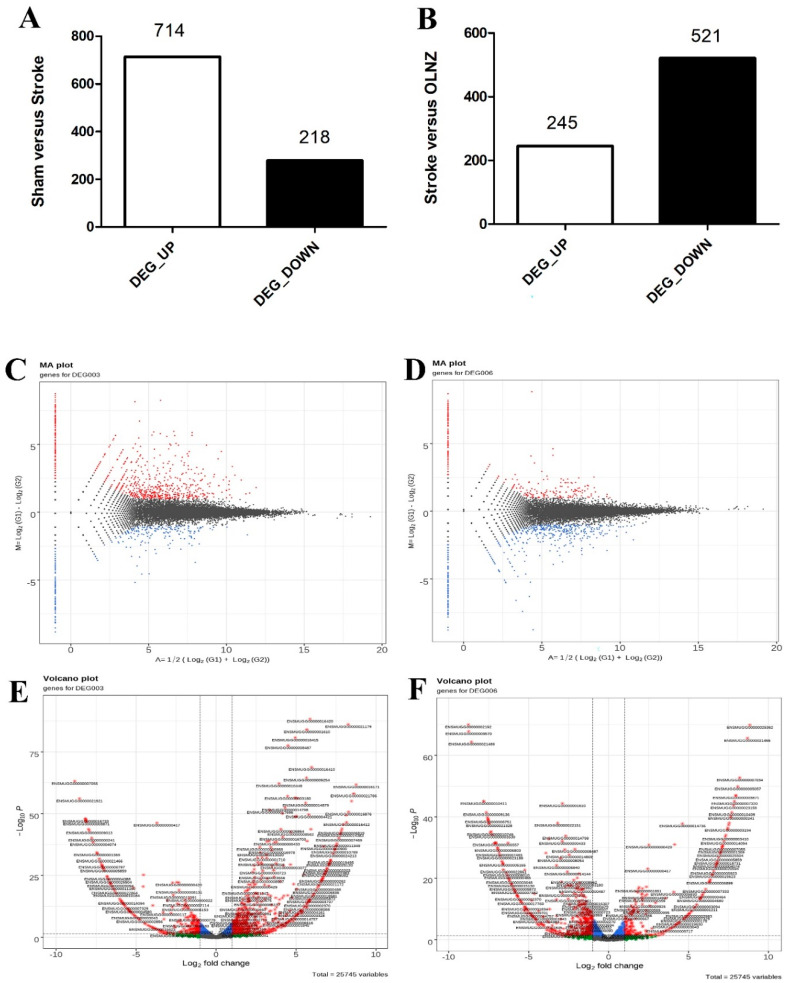
Alterations of transcriptomic gene expression in the stroke-induced hippocampus. (**A**) A total of 714 DEGs was upregulated and 218 downregulated after induction of transient ischemia (TI) using RNA sequencing (Log 2 FC); (**B**) In comparison, 245 DEGs were downregulated while 521 were upregulated in the TI-induced stroke + OLNZ treatment group; (**C**) DEGs separated (MA plot) in 1-fold change (FC) compared with the sham-TI induced group; (**D**) TI induced stroke + OLNZ treatment group; and (**E**,**F**) A volcano plot of DEGs.

The RNA sequencing was accomplished using hippocampal RNA from the sham, TI-induced, and TI-induced + OLNZ-treated groups after 5 days of cerebral ischemia. Initially, we assessed up and downregulated genes to identify DEGs among the three groups of gerbils. Bioinformatics analyses identified a total of 932 DEGs in the sham vs. TI-induced stroke hippocampus, among which 714 genes were upregulated and 218 were downregulated by TI-induced stroke (Figure 7A). In addition, 766 DEGs were identified in the TI-induced stroke vs. TI-induced stroke + OLNZ hippocampus, among which 521 genes were downregulated and 245 upregulated in the TI-induced stroke + OLNZ treatment group (Figure 7B).

We recorded a certain number of differentially upregulated RNA genes and the downregulated RNA genes of sham vs. stroke and stroke vs. OLNZ treatment group respectively (Table 2), and a certain number of differentially downregulated RNA genes and the upregulated RNA genes of sham vs. stroke and stroke vs. OLNZ treatment group respectively (Table 3) in Gene ID forms. To comprehend how DEGs are distributed across the groupings as a whole, the MA plot (Figure 7C,D) and the volcano plot (Figure 7E,F) were drawn by a threshold of log2FC > 1. Significantly elevated DEGs are highlighted in red on the MA plot, whereas significantly downregulated genes are highlighted in blue. Significant DEGs are highlighted in red and green on the volcanic plot.

### 3.6. Effects of OLNZ on Functional Pathway Involved in the Ischemic Stroke Response in the Hippocampus

**Figure 8:**
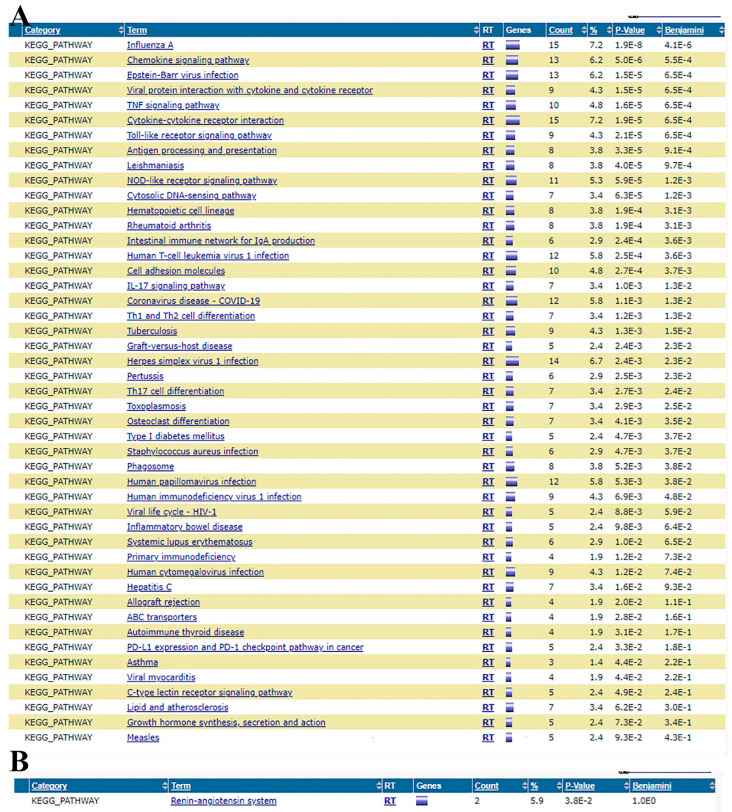
Analysis of KEGG pathways related to TI: (**A**) Functional pathways were downregulated by OLNZ treatment after TI; and (**B**) Upregulated by OLNZ treatment after TI.

The KEGG pathways in the stroke vs. OLNZ treatment group were revealed (Figure 8A,B). The analytical data showed that downregulated pathways by OLNZ treatment after induction of TI were mainly involved in chemokine and cytokine signaling.

### 3.7. Effects of OLNZ on the Expression of Complement Component mRNA

**Figure 9:**
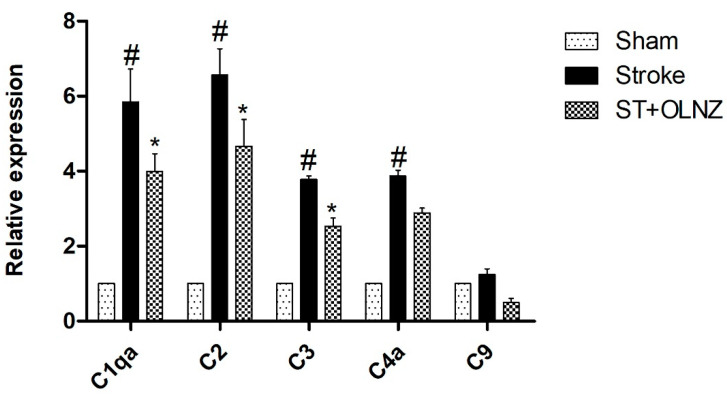
Protective role of OLNZ on gene expression of complement components in the hippocampus during transient-ischemia (TI)-induced stroke in gerbils. In TI-induced stroke, gene expression of C1q, C2, C3, C4a, and C9, was significantly upregulated, whereas treatment with OLNZ markedly downregulated the expression of these genes. Data were expressed as the mean ± SEM, *n* = 3 in each group. After performing a one-way ANOVA with Tukey’s post hoc test, *p* < 0.05 for comparisons of treatment groups with the sham group (#) and *p* < 0.05 for comparisons with the stroke group (*) were considered significant.

Based on the DEG results, we selected five genes for verification by RT qPCR. To detect the similarity of complement system activation in the hippocampus after induction of TI (Figure 9), we investigated the expression of C1q, C2, C3, C4a, and C9 mRNAs. All complement components were upregulated by ischemic insult and downregulated by TI-induced stroke + OLNZ treatment.

### 3.8. Neuroprotective Effects of OLNZ against SH-SY5Y Cell Toxicity, LDH, and ROS Release

**Figure 10:**
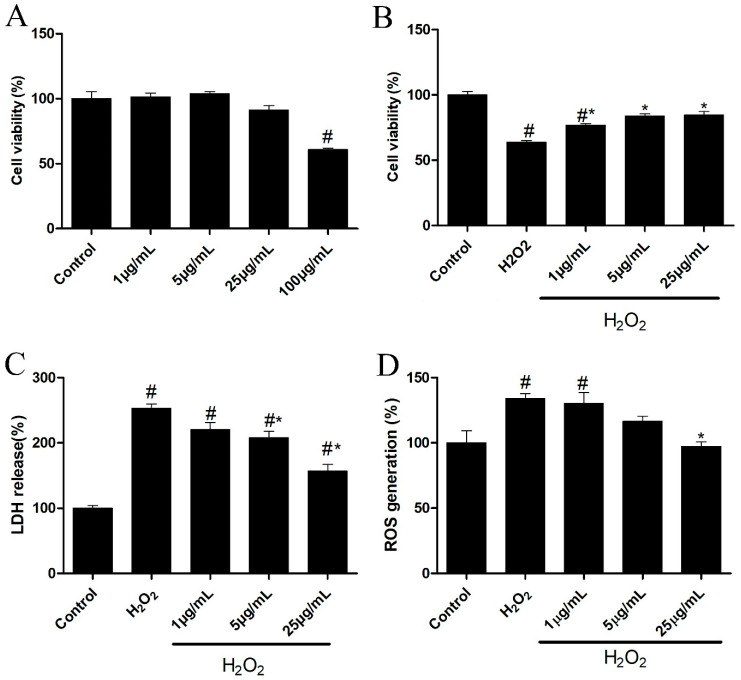
Effects of OLNZ in H_2_O_2_-induced neuronal cell death in vitro: (**A**) The non-toxic concentration of OLNZ in SH-SY5Y cells was estimated using the MTT assay; (**B**) Cell viability in H_2_O_2_-mediated toxicity was also evaluated using the MTT assay; (**C**) The release of cytotoxic marker LDH (%); and (**D**) the oxidative stress marker ROS was examined. Data were expressed as the mean ± SEM, and the experiment was repeated three times. # and * indicate a statistically significant difference compared with the control and H_2_O_2_ treatment alone, respectively, *p* < 0.05. the oxidative stress marker.

We used the MTT assay to evaluate the neuroprotective efficacy of the OLNZ using SH-SY5Y cells incubated with cytotoxic concentrations of H_2_O_2_. To identify the optimum non-toxic dose of OLNZ, SH-SY5Y cells were incubated with several concentrations of OLNZ drug (1, 5, 25, or 100 μg/mL) for 24 h. A noticeable reduction in cell viability was recorded at a high concentration of OLNZ (100 μg/mL) as related to control cultures (Figure 10A). We found that 300 μM/mL of H_2_O_2_ increased the SH-SY5Y cytotoxicity compared to control cultures. The viability of cells incubated with H_2_O_2_ increased significantly in a concentration-dependent manner in SH-SY5Y cultures co-incubated with OLNZ (1, 5, 25 μg/mL; (Figure 10B). We examined intracellular LDH and ROS release caused by H_2_O_2_ in the human neuroblastoma cell line SH-SY5Y. Our data demonstrated that H_2_O_2_ incubation greatly increased intracellular LDH and ROS formation in SH-SY5Y cells (*p* > 0.05) compared with control cultures; however, OLNZ co-incubation notably (*p* > 0.05) reduced LDH (Figure 10C) and ROS (Figure 10D) accumulation inside the cells.

### 3.9. Anti-Oxidant Activity of OLNZ in SH-SY5Y Cells

**Figure 11:**
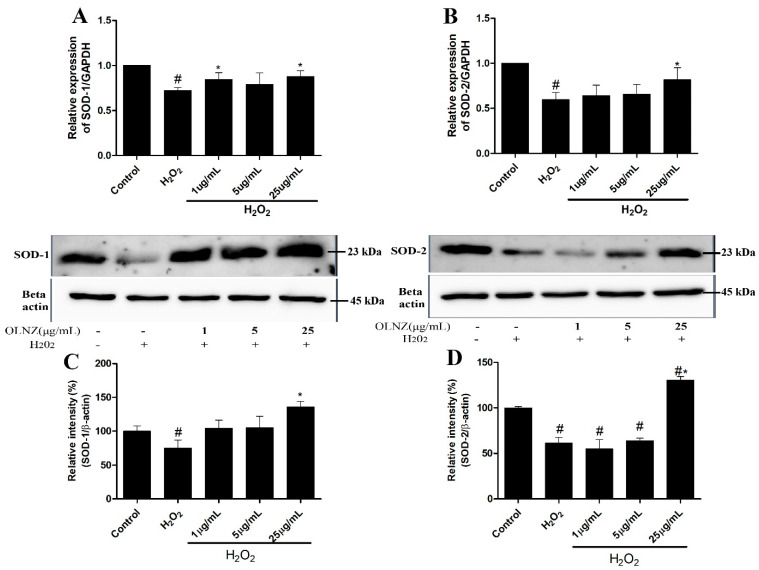
Effects of OLNZ on antioxidant enzyme (SOD-1, SOD-2) gene and protein expression in H_2_O_2_-induced SH-SY5Y cells. The expression of (%). (**A**) SOD-1 and (**B**) SOD-2 genes and (**C**) SOD-1 and (**D**) SOD-2 proteins was increased after pre-incubation with OLNZ in a concentration-dependent manner. Data were expressed as the mean ± SEM, and the experiment was repeated three times. (#) compared to the control and (*) compared to H_2_O_2_, *p* < 0.05.

We measured the expression of free radical scavenging enzyme (SOD-1 and SOD-2) genes and proteins in SH-SY5Y cells by RT-qPCR and immunoblotting analysis, respectively (Figure 11). Incubation with H_2_O_2_ stimulated oxidative stress that led to decreased expression of SOD-1 and SOD-2 mRNA (Figure 11A,B) and proteins (Figure 11C,D) compared to control cultures. In contrast, co-incubation with OLNZ and H_2_O_2_ significantly (*p* < 0.05) amplified the expression of both antioxidant enzymes.

### 3.10. Neuroprotective Effects of OLNZ to Prevent MAPK Cascade and NF-kB Activation

**Figure 12:**
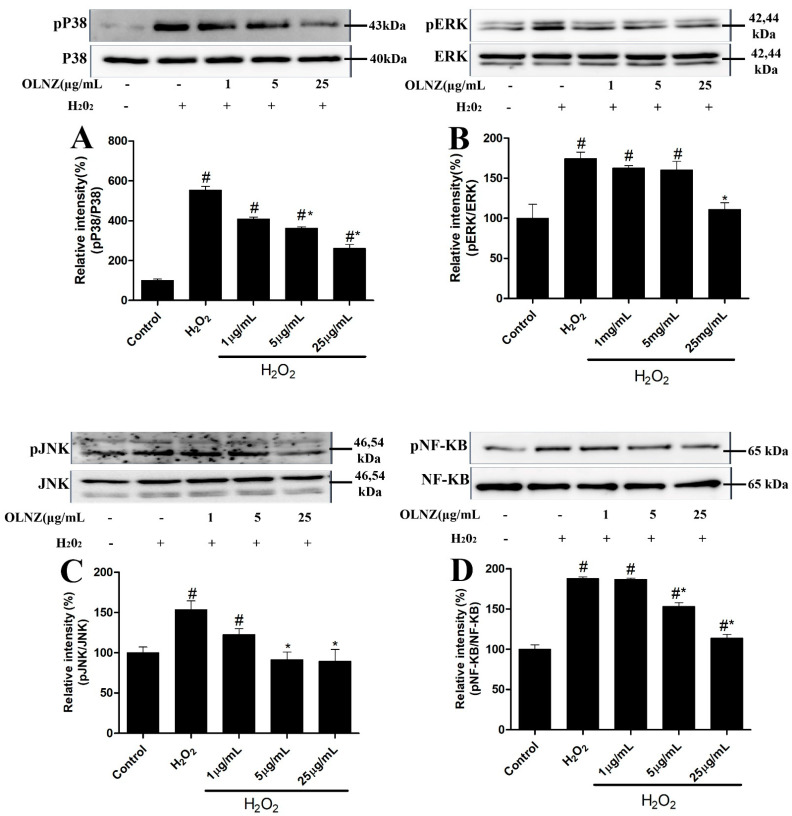
Protective role of OLNZ against the H_2_O_2_-mediated phosphorylation of ERK, JNK, p38, and NF-KB protein in SH-SY5Y cells. The expression (% of (**A**) pP38, (**B**) pERK, (**C**) pJNK, and (**D**) pNF-KB proteins decreased after pretreatment with OLNZ in H_2_O_2_-induced SH-SY5Y cells. Data were presented as the mean ± SEM, and the experiment was repeated three times. (#) compared to the control and (*) compared to H_2_O_2_, *p* < 0.05.

We found that H_2_O_2_-exposed SH-SY5Y cells elevated phosphorylation levels of MAPK (ERK1/2, JNK, and p38) (Figure 12A–C) cascade and NF-kB (Figure 12D) in comparison to the control. Meanwhile, activation of ERK, JNK, P38, and NF-kB proteins was considerably reduced (*p* < 0.05) after OLNZ pretreatment in a concentration-dependent pattern.

### 3.11. Anti-Apoptotic Effects of OLNZ in SH-SY5Y Cells

**Figure 13:**
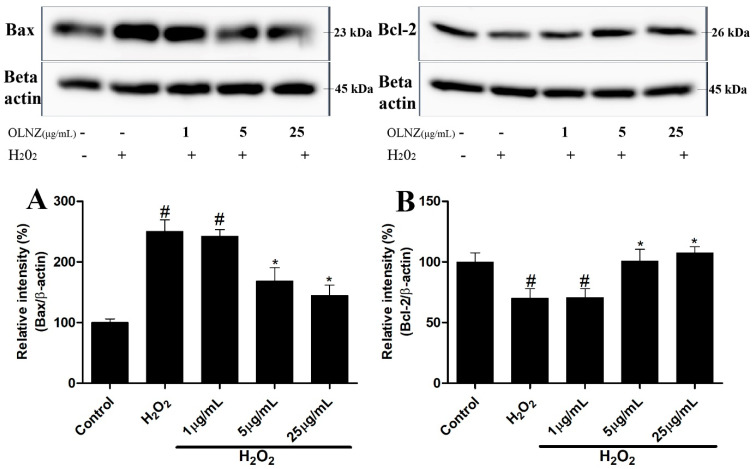
Protective role of OLNZ in H_2_O_2_-mediated apoptosis and on Bax and Bcl proteins in SH-SY5Y cells. The expression (%) of (**A**) Bax protein decreased and (**B**) Bcl-2 increased after pretreatment of SH-SY5Y cells with OLNZ in comparison with H_2_O_2_-mediated neurotoxicity in SH-SY5Y cells. All data are presented as the mean ± SEM, and the experiment was repeated three times. (#) compared to the control and (*) compared to H_2_O_2_, *p* < 0.05.

We used Western blotting to see if H_2_O_2_ and/or OLNZ had any effect on the expression of Bcl-2 and Bax proteins. As compared to the control, H_2_O_2_ considerably decreased the expression of Bcl-2 protein in SH-SY5Y cells, while significantly increasing the expression of Bax protein. Furthermore, pretreatment with OLNZ markedly (*p* < 0.05) reduced Bax and increased Bcl-2 proteins in a dose-dependent manner (Figure 13).

## 4. Discussion

During cerebral ischemia, excessive release of dopamine and serotonin induces excitotoxicity and energy deprivation and reduces blood flow in the ischemic brain, leading to irreversible brain damage [41,42,43,44]. Olanzapine is a well-known dopamine and serotonin receptor antagonist [45]. Therefore, olanzapine may be a potential neuroprotective compound; although the exact mechanism of neuroprotection by OLNZ in vivo is unclear. Further experiments are needed to investigate the actual mechanism involved. In this study, gerbils were used as an in vivo model for transient global cerebral ischemia-mediated neuronal death, and SH-SY5Y cells were used as a model for in vitro neuroprotection and potential mechanisms of action.

Transient cerebral ischemia in gerbils is brought on by blocking both common carotid arteries for 5 min [46,47,48]. In our previous study, severe neuronal cell damage was identified 5 days after induction of ischemic insult [36,49]. We also found neuronal cell death and activation of microglial cells and astrocytes in the most vulnerable part of the brain (hippocampus) after 5 days of TI. The number of CV-positive cells in the hippocampus of the OLNZ treatment group was higher as compared to the TI-induced group, but lower than in the sham group. The same pattern of NeuN immunoreactive neuronal cell expression was evident in all three groups, although the numbers of neuronal degenerative response indicator F-JB-positive cells were markedly increased in the CA1 region after induction of TI and decreased by OLNZ treatment. A previous study reported that olanzapine treatment after permanent focal cerebral ischemia in mice reduced brain damage as assessed by triphenyl tetrazolium chloride staining [34]. Other studies have revealed that oral administration of olanzapine increased the proliferation of subventricular zone neurons and decreased dendritic spine loss in rats [33,50]. Kainic acid-induced hippocampal neuronal loss in neonatal rats was also blocked by high doses of olanzapine [51]. Therefore, we can say that OLNZ treatment can prevent neuronal loss in the hippocampus induced by TI.

Many research studies have confirmed that brain ischemia, trauma, tumor growth, or neurodegenerative disease induce excessive activation of glial cells [52,53]. In ischemic insult, neuroinflammatory responses induce the secretion of various inflammatory mediators [54,55]. Our data revealed that Iba1+ microglia and GFAP+ astrocytes were significantly active across the hippocampus’s focal area in the TI-induced stroke group, in comparison with those of the OLNZ treatment and sham groups. Handi Zhang et al. showed that OLNZ alleviated astrocyte gliosis in a cuprizone (CPZ)-induced model of demyelination in C57BL/6 mice [56]. These experimental data strongly indicate that the neuroprotective effects of OLNZ are due to the modulation of TI-induced glial cell activation.

To confirm the TI-induced neuronal modification and neuroprotective effects of OLNZ, DEGs of RNA seq were evaluated. Some chemokine ligands and interferon-induced proteins (Ccl8, Cd69, Sell and Ccl7) were aggravated by induction of TI and regulated by OLNZ treatment. Ccl8 and Ccl7 are kinds of chemokines that attract monocytes to the site of trauma, infection, toxin exposure, and ischemia [57]. Cd69 triggers monocyte and NK cell activation [58] and sell recruits leukocytes in inflammatory lesions [59]. ALOX15 gene is a strong suppressor of inflammation that was downregulated by TI and upregulated by OLNZ treatment [60]. In addition, the ischemic state appeared to increase the expression of complement components that stimulate apoptosis [19,20,21]. Our qPCR data also supported a role for upregulated complement component expression in global cerebral ischemia, and that OLNZ treatment downregulated that expression. Hence, OLNZ can block the complement components mediated damage after induction of TI.

The H_2_O_2_-induced model of neurotoxicity in SH-SY5Y human neuroblastoma cells has been widely used to assess the neuroprotective effects of various compounds against oxidative stress [61,62]. Here, the same model of neurotoxicity was applied to investigate the protective effects of OLNZ, and OLNZ pretreatment was found to significantly mitigate H_2_O_2_-mediated SH-SY5Y cell death. Similarly, OLNZ pretreatment reduced LDH (a cell membrane disintegrity marker) and ROS release. Earlier experiments showed that co-incubation with OLNZ increased cell viability in β-amyloid peptide 25-35-, N-methyl-4-phenyl pyridinium ion-, and hydrogen peroxide-mediated SH-SY5Y and PC12 cell death [63,64]. So, OLNZ can regulate H_2_O_2_-induced neurotoxicity in SH-SY5Y.

Oxidative-stress-induced cytotoxicity by H_2_O_2_ is associated with increased production of ROS that damage intracellular molecules including lipids, proteins, and DNA [65,66]. Besides, TI-induced oxidative stress-mediated free radicles increase lipid peroxidation in the CA1 region of the hippocampus [40,67]. Opposed to SODs are enzymes, which stabilize most of the superoxide radicals (O_2_^−^) by oxidative stress, and thus protect cells from ROS-mediated cytotoxicity [68]. induced We investigated whether SOD-1 or SOD-2 expression was reduced by H_2_O_2_ or TI-induced oxidative stress, and whether OLNZ treatment blocked this reduction of SODs protein and gene expression. Early intervention explored pre-incubation with 10 and 100 uM/L olanzapine upregulated SOD-1 mRNA expression in PC12 cell cultures after 48 h of incubation [69]. Another experiment confirmed that the antioxidant activity of olanzapine is due to the scavenging of superoxide anions formed during the respiratory burst and augmentation of the antioxidant enzymes SODs, catalase, and glutathione peroxidase [64,70]. Therefore, OLNZ could be an important factor against oxidative stress-induced cell death.

Oxidative stress initiated by ROS production and inflammation-induced cell and tissue injuries are important factors in the activation of the MAPK cascade [71]. Activation of the MAPK cascade can be predicted based on ERK, JNK, and p38 kinase activation [72]. When the MAPK cascade is activated, it triggers apoptosis by DNA damage or caspase-8 activation [14,73]. In our study, OLNZ treatment significantly downregulated transient ischemia and H_2_O_2-_mediated phosphorylation of MAP kinases in the hippocampus and SH-SY5Y cells. Previous studies have demonstrated that olanzapine can trigger the activation of MAPKs in a dose- and time-dependent manner [30,74,75]. The phosphorylation of NF-κB is an important event in the stress response and can be triggered by a wide range of stimuli, including oxidative stress. As such, H_2_O_2_ can rapidly activate NF-κB, which plays an important part in ROS-induced apoptosis [76]. Our results indicated that OLNZ efficiently suppressed H_2_O_2_-induced phosphorylation of NF-κB. Taken together, our findings suggest that OLNZ has neuroprotective effects against oxidative stress-induced neuronal damage in both in vivo and in vitro models by regulating MAPK signaling pathway.

Bcl-2 family proteins (Bax and Bcl-2) are directly related to the mitochondrial apoptosis process [77]. These proteins control the absorbency of the mitochondrial outer membrane as well as the delivery of apoptotic mediators from the intermembranous mitochondrial region [77]. In this study, OLNZ pretreatment boosted Bcl-2 protein expression and decreased Bax protein expression in H_2_O_2_-mediated apoptosis of SH-SY5Y cells. These findings suggest that OLNZ can control apoptotic pathways that are dependent on Bax and Bcl-2.

## 5. Conclusions

In summary, OLNZ mitigates neuronal cell death and maintains almost normal hippocampal integrity. Our result authenticated that olanzapine attributed its neuroprotective impacts against TI-induced neuronal damage and H_2_O_2_-mediated neurotoxicity in SH-SY5Y cells by decreasing neuronal apoptosis, as well as oxidative stress by regulating MAPK signaling pathway. These findings provide opportunities for further research on olanzapine as a promising medicine against ischemic stroke.

## Figures and Tables

**Table 1:** Primers sequences.

Genes	Primers Name	Primer Sequence (5^′^-3^′^)	Gene Accession Number
*C1q*	C1q-F	AGGTCATCACCAACCAGGAG	XM_021631449
	C1q-R	CTTGGAGACCACTTGGAAGG	
*C2*	C2-F	CCTTGCAGAGGAGAATCTGG	XM_021655902
	C2-R	GGAGGCTTCCTTCGAGAGTT	
*C3*	C3-F	AGGTGAGGGTGGAACTGTTG	XM_021631821
	C3R	AAGGGCACGATGACATAAGG	
*C4a*	C4a-F	TCCTCCGTTCCTACAACGTC	XM_021655869
	C4a-R	CGTTGGCTTCCCTTGTGTAT	
*C9*	C9-F	TGTAAACATCACCCGCGATA	XM_021654489
	C9-R	CAACGGTCTTGGCTTCTCTC	
*GAPDH*	GAPDH-F	AGAACATCATCCCTGCATCC	XM_021636934
	GAPDH-R	GATCCACGACAGACACGTTG	
*SOD-1*	SOD-1-F	AGGCCGTGTGCGTGCTGAAG	NM_000454
	SOD-1-R	CACCTTTGCCCAAGTCATCTGC	
*SOD-2*	SOD-2-F	CTGCTCCCCGCGCTTTCTTA	NM_001024466
	SOD-1-R	CACGTTTGATGGCTTCCAGC	
*GAPDH*	GAPDH-F	TTCACCACCATGGAGAAGGC	NM_001357943
	GAPDH-R	GGCATGGACTGTGGTCATGA	

**Table 2:** List of the few most upregulated protein-coding genes in the transient-ischemia (TI)-induced stroke model in gerbils, and the few most downregulated protein-coding genes in the TI-induced stroke + olanzapine treatment group.

Gene Symbol	Differentially Expressed Genes Upregulated by Stroke (Log2FC)	Symbol of Genes	Differentially Expressed Genes Downregulated by Stroke+ OLNZ Treatment (Log2FC)
Cxcl10	8.26	Ccl8	−6.29
Ccl5	7.43	Cd69	−5.24
Ccl2	7.43	Ciita	−5.09
Ccl8	7.32	Ccl7	−4.81
Cd69	6.85	AA467197	−4.79
Oasl1	6.73	Sell	−4.64
Cxcl11	6.53	Mrgbp	−4.58
Sell	6.25	Ccr7	−4.53
Ifit1	5.97	Cxcl13	−4.41
Ifit2	5.88	A930017K11Rik	−4.37
Ccl7	5.84	Msr1	−4.09
Isg15	5.80	Slamf9	−4.05
Ccl12	5.69	Tmprss13	−3.88
Hcar2	5.67	Lcn2	−3.84
Il1b	5.67	Cks2	−3.79
Ttr	5.65	Cplx3	−3.74
Oas2	5.60	Slc30a2	−3.69
Rsad2	5.48	Fzd10	−3.58
Gpr171	5.08	Neurl3	−3.58
Hk3	5.04	Nkg7	−3.53
C1qa	2.04	C1qa	−0.64
C2	2.88	C2	−1.09
C3	1.68	C3	−0.83
C4b	1.82	C4b	−0.04
C9	0.09	C9	0

**Table 3:** List of the few most downregulated protein-coding genes in the transient-ischemia (TI)-induced stroke model in gerbils, and the few most upregulated protein-coding genes in the TI-induced stroke + olanzapine treatment group.

Gene Symbol	Differentially Expressed Genes Downregulated by Stroke (Log2FC)	Symbol of Genes	Differentially Expressed Genes Upregulated by Stroke + OLNZ Treatment (Log2FC)
Eif2s3y	−7.93	Eif2s3y	5.21
Rec8	−3.26	AGTR1	3.44
1700067K01Rik	−3.26	Mlph	2.70
Nyx	−3.26	Fam71d	2.58
Kcnj15	−3.26	Fabp4	2.44
Kcnk3	−3.26	Slc7a9	2.34
Nanos1	−3.09	Prr29	2.32
Dmrta1	−3.09	Rec8	2.12
Ccdc71l	−3.09	Slc5a7	1.99
Alx3	−3.09	Ttll9	1.97
Tbx2	−2.90	Rerg	1.80
Alx1	−2.67	Dcdc2b	1.70
Cnr2	−2.67	Alox15	1.70
Mpo	−2.67	Tctex1d1	1.70
Cfap157	−2.59	Cavin3	1.70
Slc4a1	−2.41	Tmc5	1.60
Icam4	−2.20	Susd1	1.49
St8sia2	−2.09	Ppbp	1.49
Nphs1	−1.93	Crhr2	1.47
Alox15	−1.85	Det1	1.44

## Data Availability

All experimental data produced during this investigation has been inserted in this main manuscript.
